# AplusB: A Web Application for Investigating A + B Designs for Phase I Cancer Clinical Trials

**DOI:** 10.1371/journal.pone.0159026

**Published:** 2016-07-12

**Authors:** Graham M. Wheeler, Michael J. Sweeting, Adrian P. Mander

**Affiliations:** 1 MRC Biostatistics Unit Hub for Trials Methodology Research, Cambridge Institute of Public Health, Cambridge, United Kingdom; 2 Cancer Research UK and UCL Cancer Trials Centre, University College London, London, United Kingdom; 3 Cardiovascular Epidemiology Unit, Strangeways Research Laboratory, University of Cambridge, Cambridge, United Kingdom; National Cancer Center, JAPAN

## Abstract

In phase I cancer clinical trials, the maximum tolerated dose of a new drug is often found by a dose-escalation method known as the *A* + *B* design. We have developed an interactive web application, *AplusB*, which computes and returns exact operating characteristics of *A* + *B* trial designs. The application has a graphical user interface (GUI), requires no programming knowledge and is free to access and use on any device that can open an internet browser. A customised report is available for download for each design that contains tabulated operating characteristics and informative plots, which can then be compared with other dose-escalation methods. We present a step-by-step guide on how to use this application and provide several illustrative examples of its capabilities.

## Introduction

In oncology, phase I trials are the first experimentation of a new drug in humans. They are primarily concerned with assessing how safe a drug is and aim to identify the drug’s Maximum Tolerated Dose (MTD). Definitions of the MTD vary; the National Cancer Institute (NCI) defines the MTD as “the highest dose of a drug or treatment that does not cause unacceptable side effects” [1], whereas in statistical literature the MTD is defined as “the dose expected to produce some degree of medically unacceptable, dose-limiting toxicity (DLT) in a specified proportion of patients (e.g. 30%)” [2]. What is classed as a DLT will depend on the trial being conducted, but it is often defined as at least one grade 3 or higher toxicity as per the NCI Common Terminology Criteria for Adverse Events (CTCAE) [3]. Phase I oncology trials are usually dose-escalation studies, where the dosage given to a cohort of patients increases if the previous cohort tolerated their treatment; this continues either until the number of DLTs experienced in the current cohort inhibit further escalation, or the maximum sample size is reached. Rule-based designs, where fixed rules applied to counts of DLT/non-DLT responses govern the escalation and de-escalation of doses, have long been popular with clinicians in phase I trials of cytotoxic treatments [4, 5]. The appeal of rule-based designs is that they do not require the dose-toxicity relationship to be modelled according to some function of dose, and that dose escalation decisions are entirely prescriptive.

One family of rule-based designs used for phase I trials is *A* + *B* designs [6], which includes the commonly used 3 + 3 design [4, 7]. In general, small cohorts of patients receive a dosage of a new treatment and the number of patients in the cohort that experience a DLT determines whether the next cohort receive a higher, equal, or lower dosage than the current cohort. Several papers [6, 8–10] have documented analytical studies of general *A* + *B* designs and offered different operating characteristics relevant to phase I clinical trials. Lin and Shih [6] derived several statistical formulae for the operating characteristics of *A* + *B* designs, both with and without dose de-escalation. These formulae are implemented in the S-Plus program pmtd (code available from author upon request), which calculated the expected sample size, the average number of DLTs per dose level, the probability of recommending each dose as the MTD and an approximation of the probability of assigning patients to each dose; however, they did not derive the exact distribution for the trial sample size, or the distribution of the percentage of trial patients experiencing a DLT. Reiner *et al*. [8] wrote a PASCAL program for the 3 + 3 design to calculate the probability of recommending a dose as the MTD conditional on the trial stopping after different numbers of participants, the experimentation percentages at each dose and also the expected number of patients in a trial. Kang and Ahn [9] used a FORTRAN 77 program to determine the exact distribution of the MTD for the 3 + 3 design over a set of pre-determined doses, given probabilities of DLT at each dose level. However, information regarding the dose allocation and DLT outcomes of each cohort that would allow exact distributions/probabilities for other relevant quantities to be calculated was discarded. Much of the past work in exploring the operating characteristics of the 3 + 3 design has either been lost since publication, or presented a limited set of operating characteristics.

Whilst we do not necessarily advocate using *A* + *B* designs for dose-escalation studies, it is important that their operating characteristics can be clearly, easily and quickly summarised because of their ubiquity in clinical practice. The objective of this paper is to provide a tool that comprehensively summarises *A* + *B* designs so that they may be compared with other dose-escalation methods and aid the trial design process. The underlying programs in our web-based application, *AplusB*, determine all possible trials for a particular *A* + *B* design (with or without dose de-escalation) and are based on those from the threep3 program (available in the R package bcrm [11]), which only offers operating characteristics for the 3 + 3 design with dose de-escalation [12]. We describe the *AplusB* application and how it is used in the Methods section. In the Results section we investigate the operating characteristics of different designs applied to four example dose-toxicity scenarios and show the outputs that the user is provided with. We conclude with a summary of the *AplusB* application and its potential use in clinical practice.

## Methods

The *AplusB* application is written in the R programming language [13] and made freely available using the Shiny package [14]. To access the application online, visit http://www.mrc-bsu.cam.ac.uk/software and view the *Web applications* subsection. The R code for *AplusB* can be downloaded from GitHub at https://github.com/graham-wheeler/AplusB.

### The *A* + *B* design

**Fig 1:**
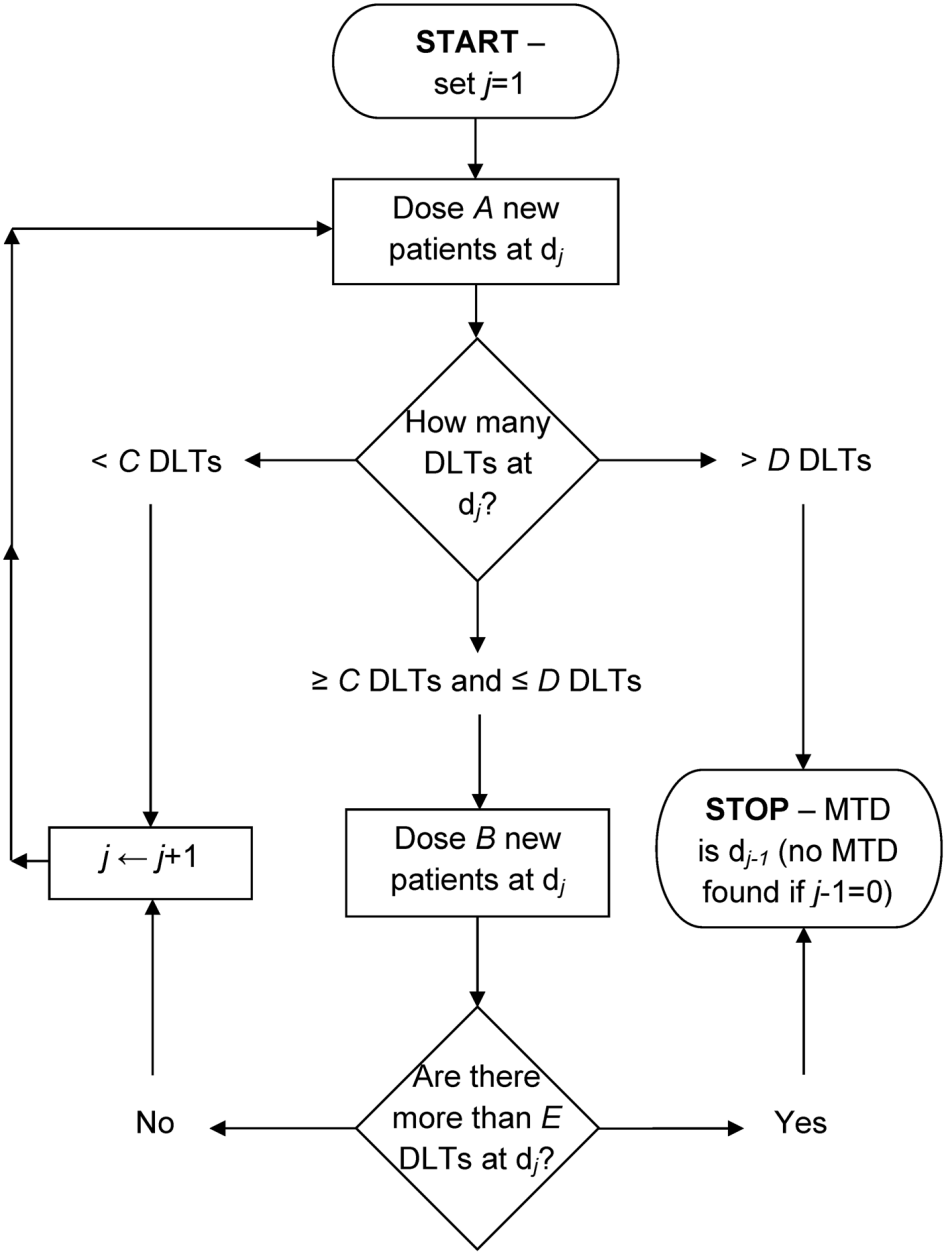
Design schematic of the *A* + *B* design without dose de-escalation.

**Fig 2:**
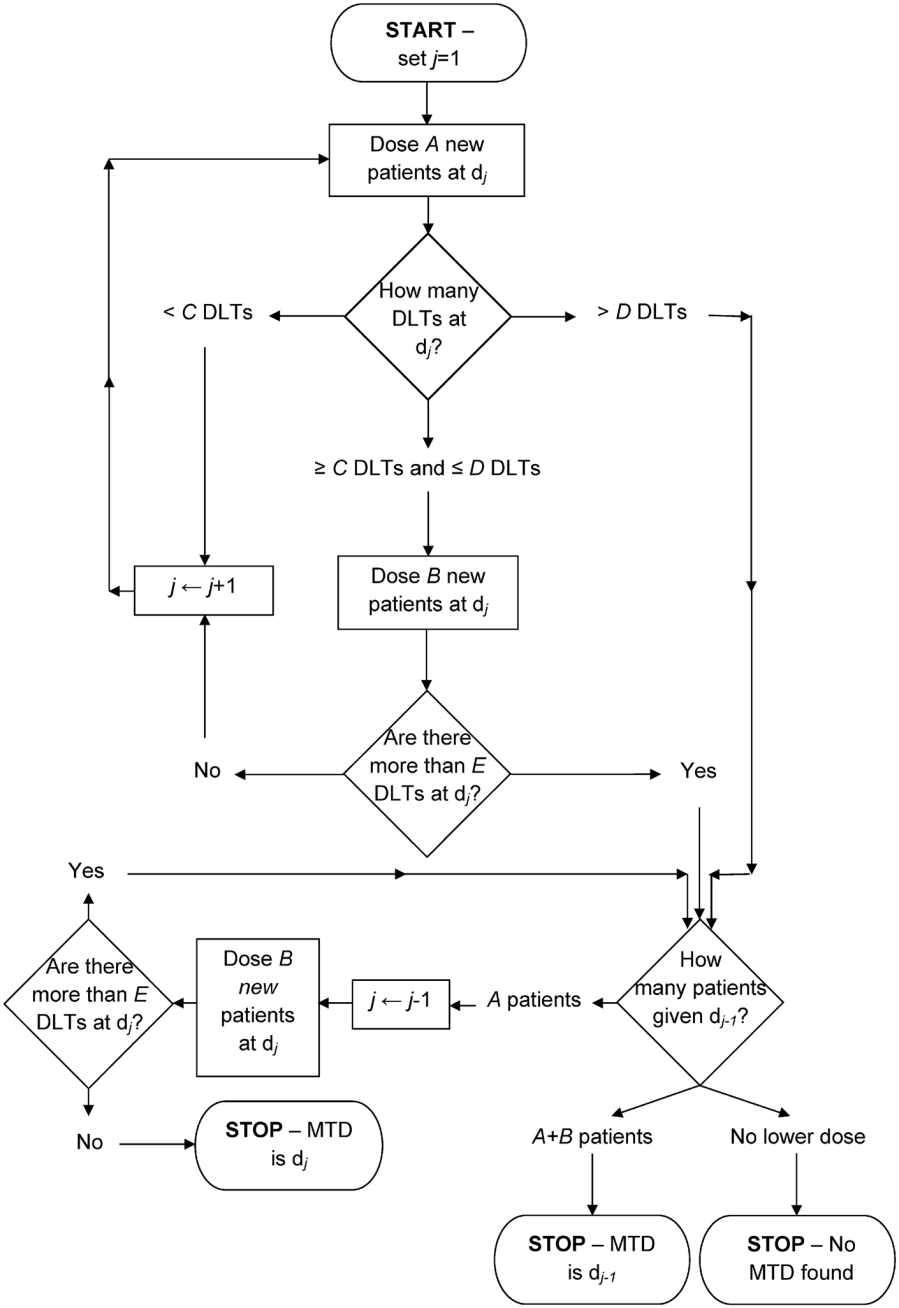
Design schematic of the *A* + *B* design with dose de-escalation.

The *A* + *B* design is a rule-based approach for conducting dose-escalation studies; the 3 + 3 design [4, 7] is one specific sub-design that is implemented in the vast majority of phase I cancer clinical trials [5, 15–17]. The *A* + *B* design obtains its name from *A* and *B*, two positive integers that represent the number of potential patients at a given dose level. The full specification of the design requires six parameters; integers *A*, *B*, *C*, *D* and *E*, and an indicator of whether dose de-escalation is permitted or not. Specifically, parameters *A* − *E* are defined as follows: *A* is the number of patients in the first cohort that are assigned to a dose; *B* is the number of patients in the second cohort (if required) that are assigned to a dose; *C* is the minimum number of DLTs needed out of *A* patients to assign *B* more; *D* is the maximum number of DLTs needed out of *A* patients to assign *B* more, otherwise the trial stops/de-escalates; *E* is the maximum number of DLTs out of *A* + *B* patients allowed so that the dose may be escalated for the next cohort of *A* patients. The decision trees that the *A* + *B* design follows, both without and with dose de-escalation, are best shown as flow charts (Fig 1; Fig 2). In general, if the number of patients experiencing a DLT at a particular dose level is below a lower bound, then the dose is increased for the next cohort; if it is above an upper bound, the trial is stopped or dose de-escalated, and if it lies between these bounds, the next cohort of patients receive the same dose level. The constants {*A*, *B*, *C*, *D*, *E*} can be replaced with {3, 3, 1, 1, 1}, with dose de-escalation not permitted, to obtain the traditional 3 + 3 design [4, 7].

### Using the application

**Fig 3:**
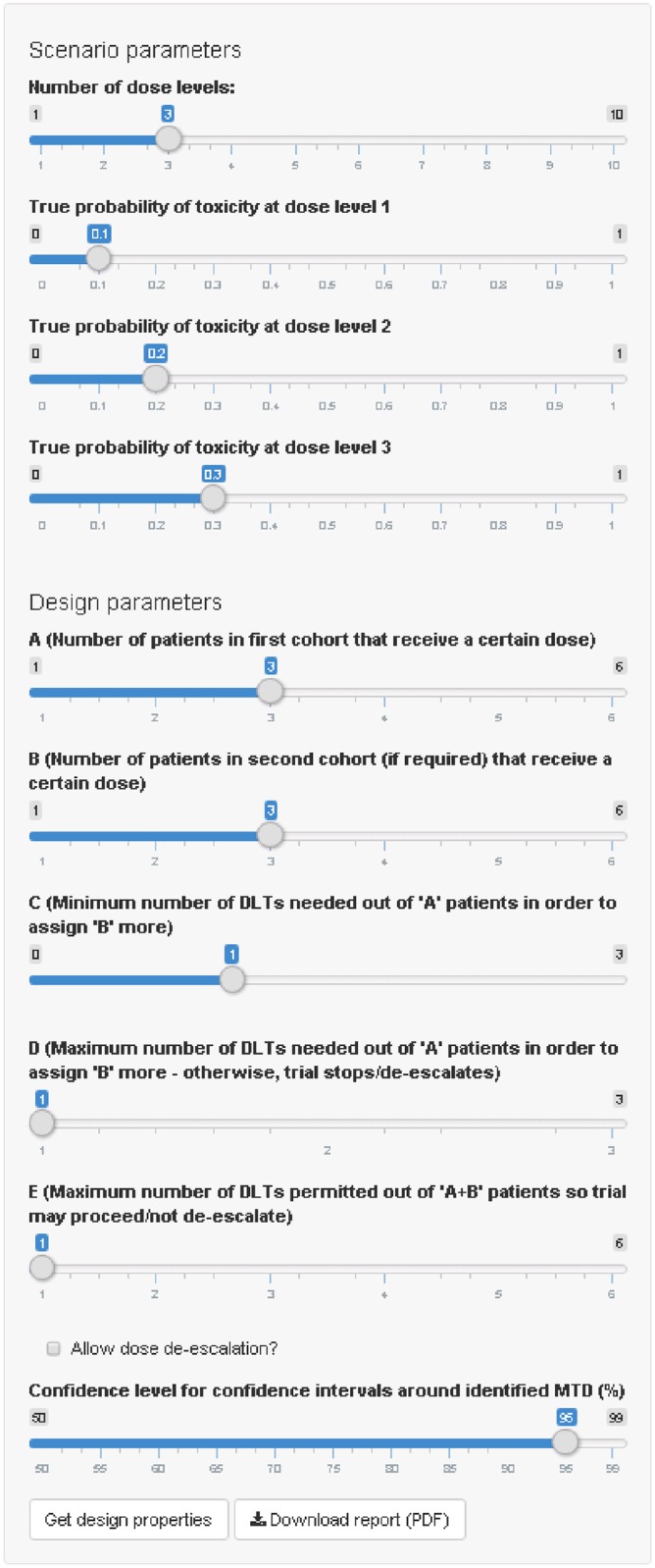
Graphical user interface (GUI) for scenario and design parameters.

**Fig 4:**
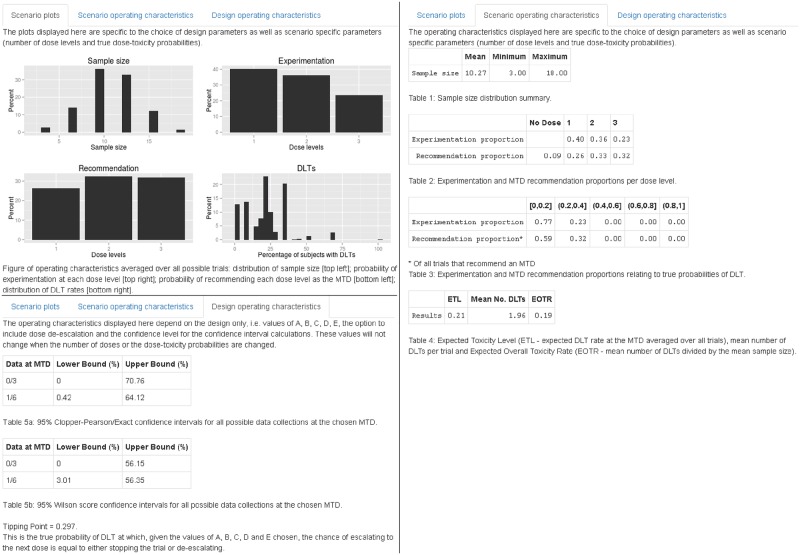
Output tabs for *AplusB* application. The *Scenario plots* tab shows the performance of four operating characteristics. The *Scenario operating characteristics* tab provides tabular and numerical summaries of key operating characteristics that depend on the DLT probabilities chosen on the left-hand panel. The *Design operating characteristics* tab shows operating characteristics dependent only upon the design selected, specifically confidence intervals and the *tipping point*.

The application has a simple graphical user interface (GUI), where the user specifies *Scenario parameters* and *Design parameters* to control the set-up of the trial (Fig 3). The scenario parameters to be specified are the number of dose levels and the true probabilities of DLT for each dose. Changing the number of dose levels will automatically change the number of sliders available to specify true DLT probabilities. The design parameters to be specified are: the integers {*A*, *B*, *C*, *D*, *E*}, which determine the rules for escalation, de-escalation (if permitted) and trial termination; the choice of dose de-escalation (via a checkbox); and the confidence level for confidence intervals for the estimated DLT probability at the selected MTD. After these have been specified, clicking the *Get design properties* button will begin the computations and display the results in the three available output tabs: Scenario plots; Scenario operating characteristics; and Design operating characteristics (Fig 4).

The *Scenario plots* tab shows four bar charts: the sample size distribution (top left); experimentation percentages at each dose level (top right); MTD recommendation percentages at each dose level (bottom left); and the distribution of DLT rates (bottom right). The *Scenario operating characteristics* tab provides a summary of the sample size distribution, experimentation and MTD recommendation percentages for each dose level, and for different intervals of true DLT probability (specifically [0, 0.2], (0.2, 0.4], (0.4, 0.6], (0.6, 0.8], and (0.8, 1]). This tab also provides the Expected Toxicity Level (ETL), the mean number of DLTs and the Expected Overall Toxicity Rate (EOTR), all of which we define in the Operating characteristics subsection. The *Design operating characteristics* tab provides two types of confidence interval for empirical data collections that are to be found at the MTD identified in a trial; for example, in a 3 + 3 trial without dose de-escalation, the trial will end with either no DLTs out of three patients at the MTD, or one DLT out of six patients. Also provided is a new operating characteristic called the *tipping point* of the design, which we define in the following subsection and discuss further in S1 Text.

### Operating characteristics

**Fig 5:**
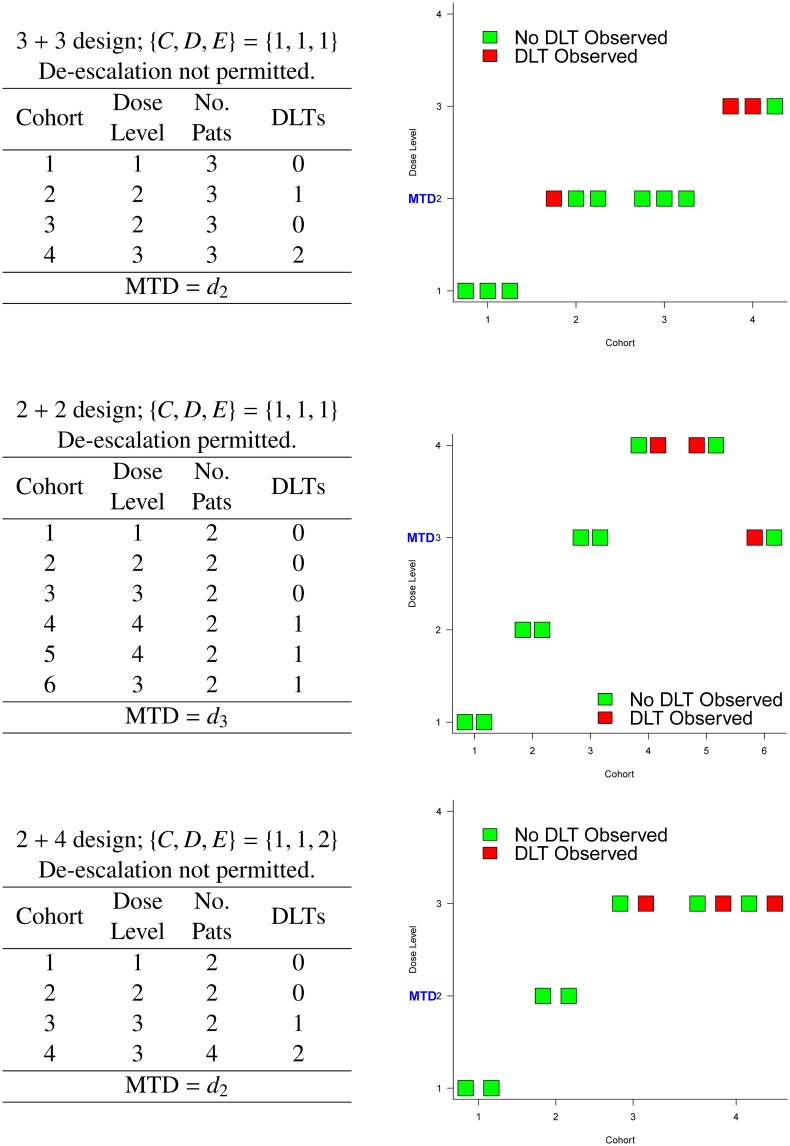
Example trial pathways for various *A* + *B* designs.

Given an *A* + *B* design specified by {*A*, *B*, *C*, *D*, *E*}, the dose de-escalation indicator and *J* dose levels denoted by *d*_*j*_ (*j* = {1, …, *J*}), we can calculate several operating characteristics of interest. The operating characteristics provided by *AplusB* consider each possible *trial pathway*, defined as the sequence of dose levels each cohort receives, along with the number of patients and number of DLTs per cohort, and the probability of each trial pathway occurring (Fig 5). Each trial pathway is unique, and some trial pathways are more likely to occur than others (e.g. a scenario of five dose levels with a large true probability of DLT at dose level 1 is more likely to lead to experimentation at dose level 1 only rather than escalate all the way to dose level 5). Therefore, the chance of every possible trial pathway occurring, no matter how remote, should be correctly incorporated into operating characteristic calculations. For a particular *A* + *B* design and dose-toxicity scenario, the distribution of trial pathways is used by *AplusB* to accurately compute operating characteristics; in the “Comparison to pmtd program” subsection, we explore how the calculations of experimentation percentages in *AplusB* differ to those in another program, pmtd. For an *A* + *B* design with true DLT probabilities specified, *AplusB* computes the sample size distribution, MTD recommendation probabilities and experimentation probabilities per dose level, the ETL (the expected probability of DLT at the MTD) and EOTR (the expected proportion of patients experiencing DLTs), and the distribution of DLT rates. Furthermore, regardless of the true DLT probabilities specified, *AplusB* also provides confidence intervals (the level of confidence can be chosen by the user) for the estimated probability of DLT given possible empirical data obtained at the dose chosen as the MTD at the end of the trial, and a new design operating characteristic called the *tipping point*. We provide formulae for these operating characteristics in S1 Text.

The *tipping point* is the true DLT probability a dose must have at which the chance of escalating to the next dose level is equal to the chance of not escalating (i.e. de-escalating or stopping the trial). For an *A* + *B* design with de-escalation permitted, we can say that the probability of choosing a dose with true probability of DLT above the tipping point will be at most 50%, i.e. we are more likely to select one of the doses with true probability of DLT below the tipping point. However, for an *A* + *B* design with de-escalation not permitted, we can say that the modal MTD (that is, the dose most likely to be chosen as the MTD) will have a probability of DLT less than or equal to the tipping point (see S2 Text for proof). This implies that for the classical 3 + 3 design with {*C*, *D*, *E*} = {1, 1, 1} and dose de-escalation not permitted, which has a tipping point of 0.297, the dose most likely to be chosen as the MTD will in fact have a true probability of DLT less than or equal to 29.7%, and not 33% that is often assumed [18].

### Downloadable report

Once design properties have been calculated and presented in the browser window, the user may click on the *Download report (PDF)* button in the bottom-left of the screen. This produces a two-page PDF report with a summary of the design chosen, design and scenario operating characteristics, and a plot of the main operating characteristics shown in the browser window. This allows a record of evaluated designs to be stored, presented and subsequently compared to operating characteristics from other dose-escalation designs if required.

## Results

We now examine four examples of dose-toxicity scenarios, each of which will be evaluated by a particular *A* + *B* design. We use a 3 + 3 design with dose de-escalation not permitted to evaluate examples I and II, and a 3 + 3 design with dose de-escalation permitted to evaluate example III. For example IV, we use a 2 + 4 design with dose de-escalation permitted.

**Fig 6:**
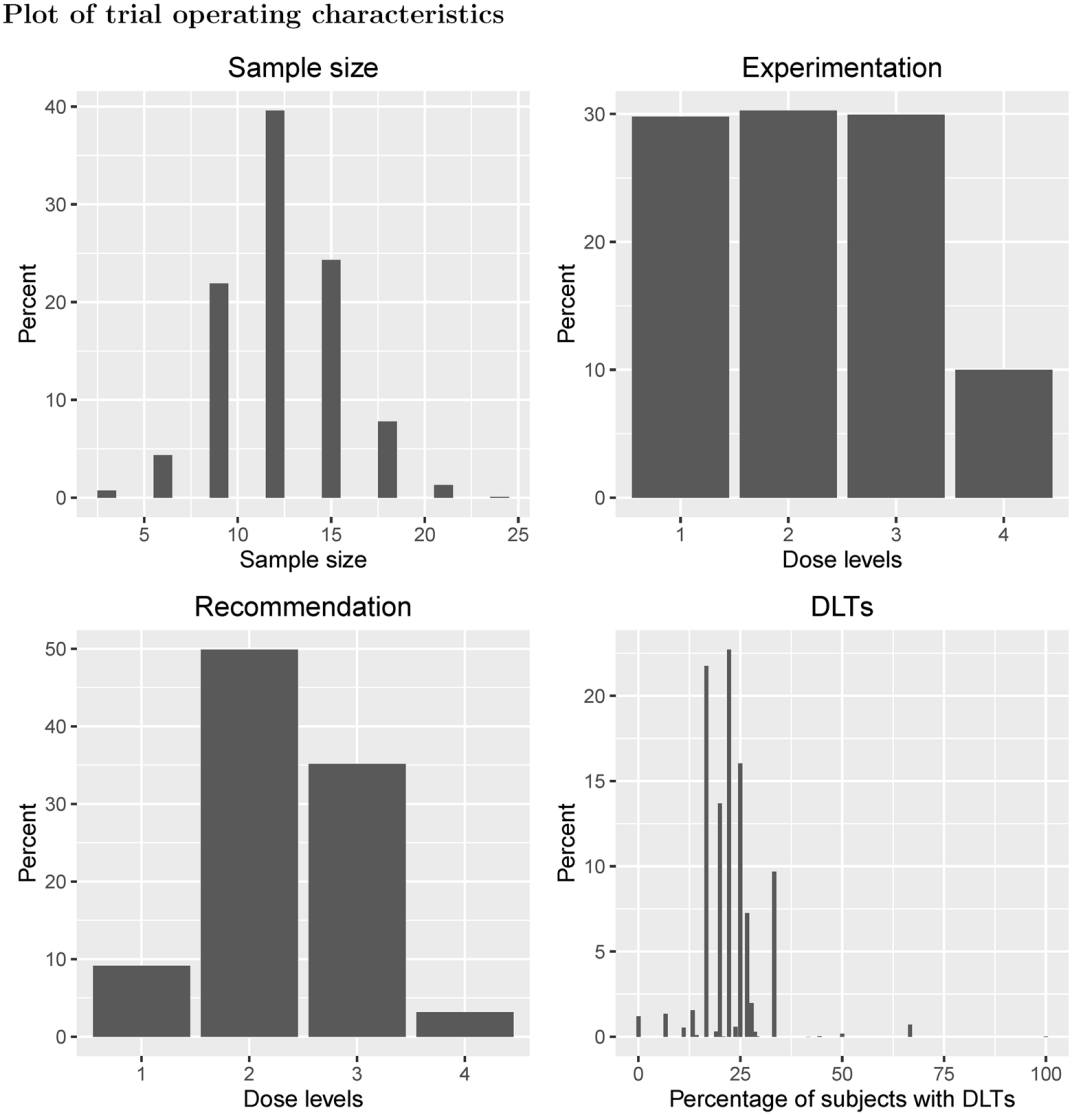
Scenario plots for Example I.

Example I shows a traditional 3 + 3 design applied to four dose levels with true DLT probabilities equal to {0.05, 0.10, 0.33, 0.60} (Fig 6). The dose chosen as the MTD has either zero DLTs out of three patients observed, or one DLT out of six patients. Using these data, the 95% Clopper-Pearson confidence intervals for the estimated probability of DLT at the MTD are (0%, 70.76%) and (0.42%, 64.12%) respectively. Dose level 2 has a 50% chance of being chosen as the MTD, despite having a true DLT probability of 10%. Furthermore, there is a 3% chance that no dose is selected as the MTD due to safety reasons, despite the lowest dose only having a 5% chance of causing a DLT. There is a 10% chance that patients will receive the highest dose, which has a 60% chance of causing a DLT. On average 22% of patients will experience a DLT (EOTR) and the ETL is 19%, much lower than the 33% that is often assumed in phase I trials using the 3 + 3 design.

**Fig 7:**
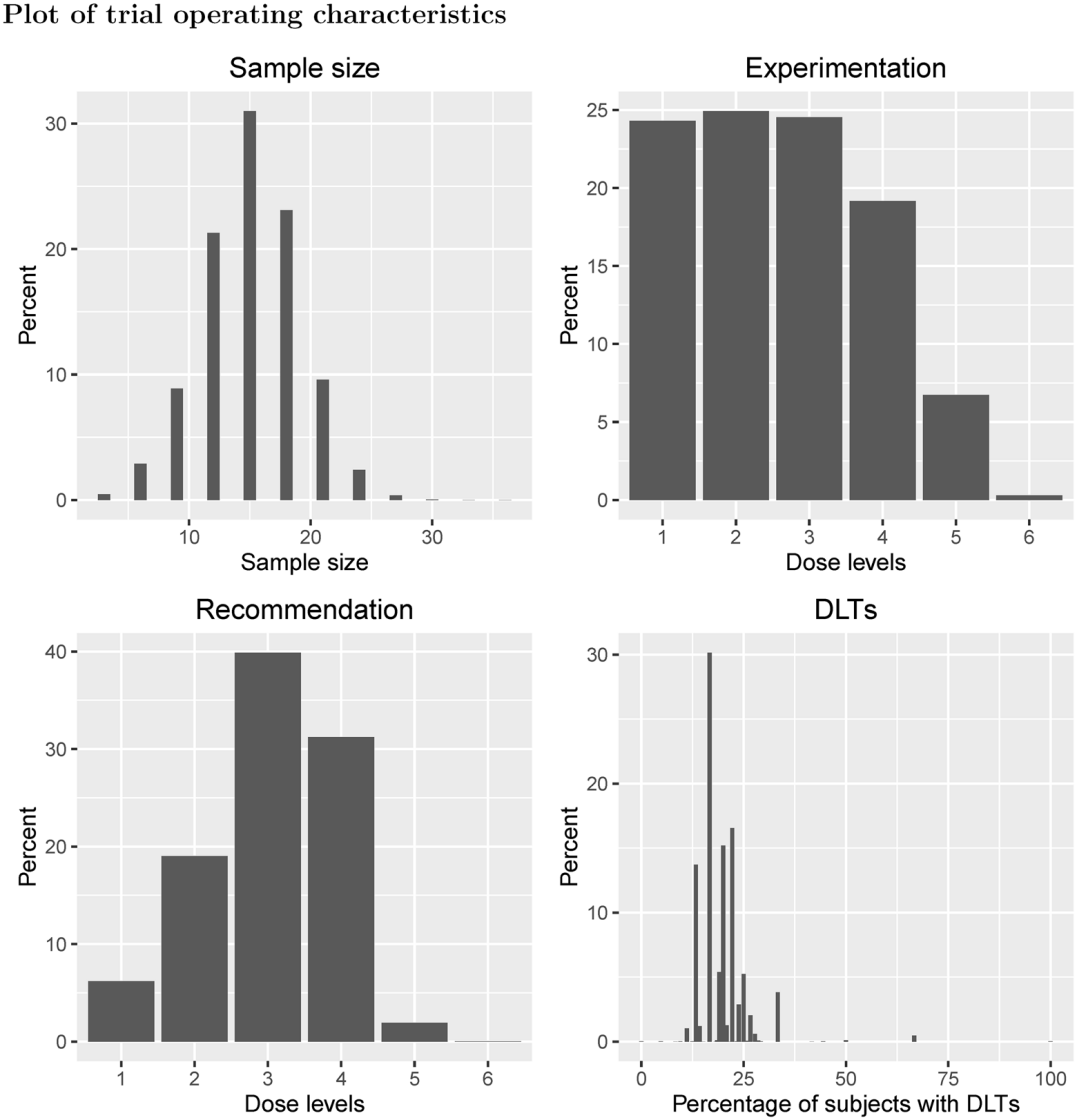
Scenario plots for Example II.

Example II shows a traditional 3 + 3 design applied to six dose levels with true probability of DLT equal to {0.04, 0.08, 0.16, 0.32, 0.64, 0.80} (Fig 7). In this scenario, the chances of selecting dose levels 3 and 4 as the MTD, which have true probabilities of DLT equal to 16% and 32%, are 40% and 31% respectively. Although three dose levels have DLT probabilities of at most 16%, there is a 2% chance that no MTD will be found for safety reasons. Furthermore, there is only a 19% chance that dose level 4 will be given to patients, with a 7% chance that dose level 5, with true DLT probability of 64%, will be given to patients. Approximately 19% of patients will experience DLT and the ETL under this scenario is 20%. For both examples I and II, the design’s tipping point is 0.297, i.e. the dose most likely to be chosen as the MTD will have a true DLT probability less than 0.297.

**Fig 8:**
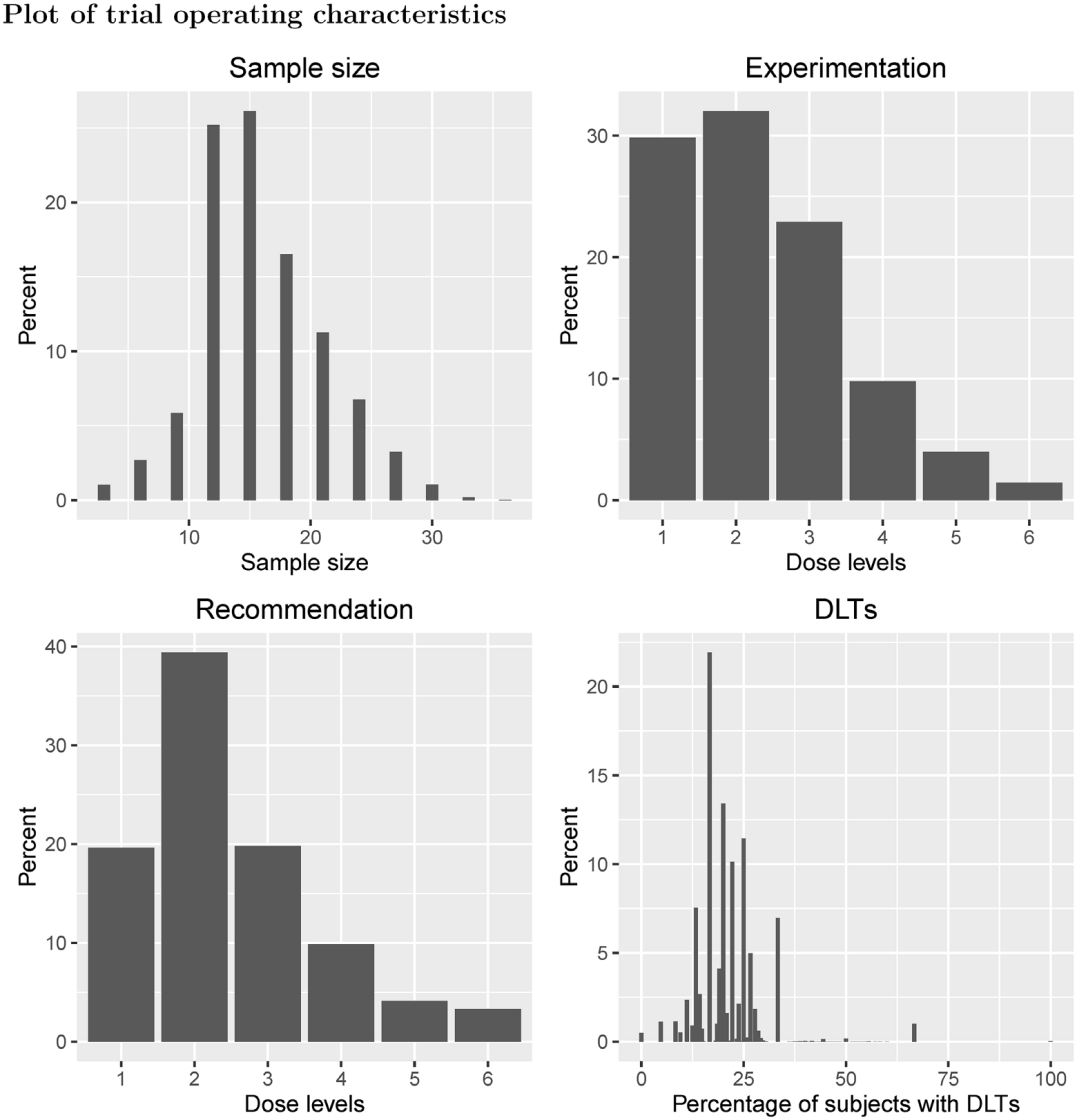
Scenario plots for Example IV.

In example III, six doses were investigated in a 3 + 3 design with dose de-escalation permitted, with true DLT probabilities {0.06, 0.15, 0.29, 0.31, 0.33, 0.35} (Fig 8). For this scenario, the DLT probabilities plateau around the suspected target toxicity level of 33% (dose level 5). Under this design, if an excessive number of DLTs are observed at the current dose level (at least two DLTs out of three or six patients) and only three patients have received the dose level immediately below, then the next three patients will be given the dose level immediately below. With respect to MTD recommendation, there is only a 4% chance that dose level 5 is chosen as the MTD at the end of the trial, and dose level 2 is the most likely dose to be chosen as the MTD (39%). The 95% Clopper-Pearson confidence intervals for the estimated probability of DLT at the MTD, at which we will have seen either zero or one DLT out of six patients, are (0%, 45.93%) and (0.42%, 64.12%) respectively. Similar to example I, the ETL is 19% and the EOTR is 20%. For experimentation, there is only a 4% chance that patients will be given dose level 5, and a 62% that patients will receive dose levels 1 or 2. Since the dose-toxicity curve becomes flat for the last four doses, the 3 + 3 design used here is unable to identify dose level 5 (or dose level 4, with true DLT probability of 0.31) with a suitable level of certainty, and most patients will only receive the two lowest dose levels and not higher levels that are still considered tolerable.

**Fig 9:**
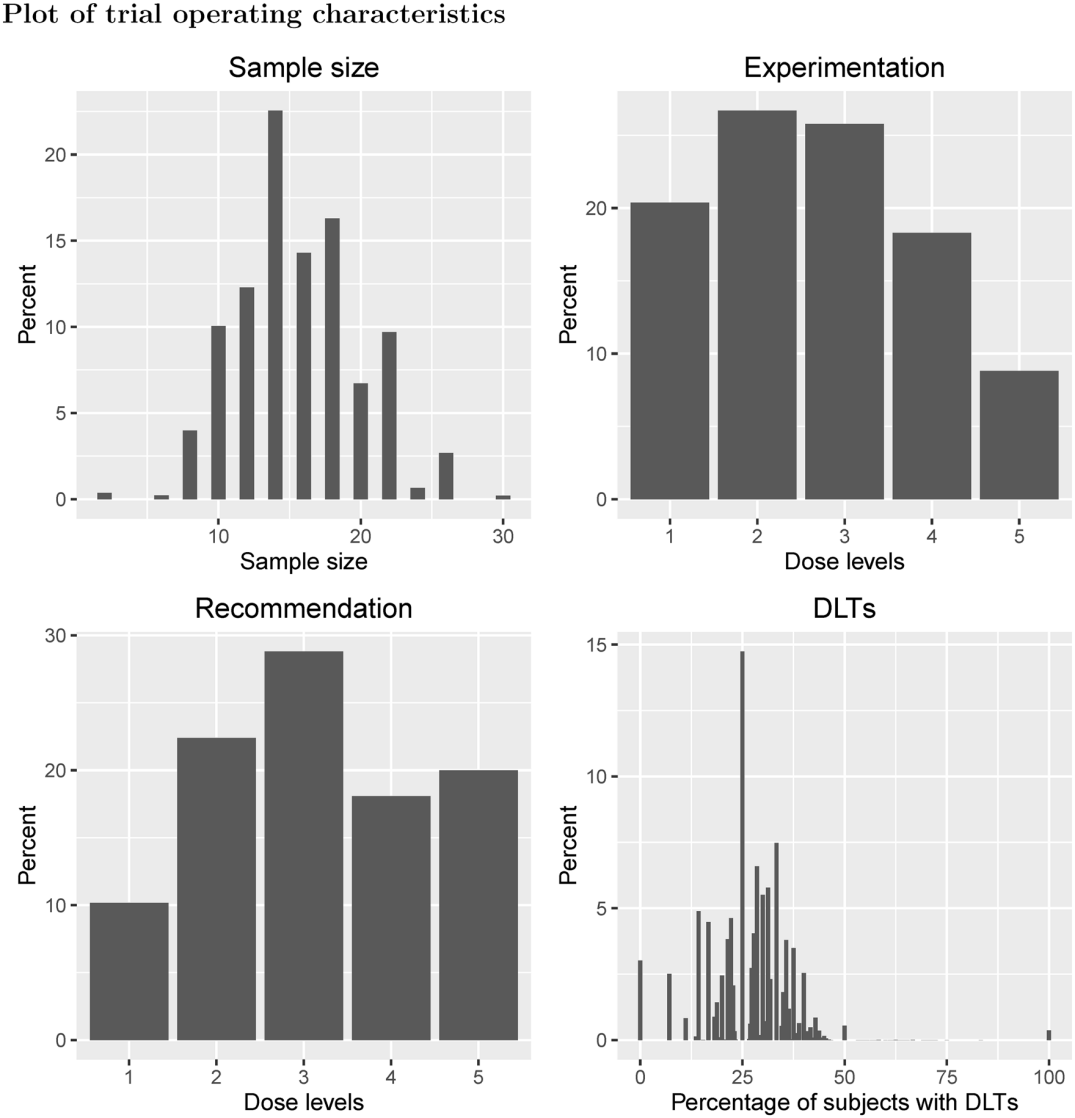
Scenario plots for Example IV.

Example IV evaluates a 2 + 4 design with {*A*, *B*, *C*, *D*, *E*} = {2, 4, 1, 1, 2} and permits dose de-escalation before stopping the trial; this design is applied to five dose levels with true DLT probabilities equal to {0.06, 0.20, 0.30, 0.40, 0.45} (Fig 9). Under this design, if no patients in the two-patient cohort have DLTs, the dose is escalated. If one patient out of two has a DLT, then four more patients are given the current dose, and if both patients in the two-person cohort have a DLT, the dose is de-escalated to the previous level. When four extra patients are given a particular dose, if two or fewer patients out of six experience DLT, then the dose is escalated, otherwise the dose is de-escalated. The 95% Clopper-Pearson confidence intervals for the probability of DLT at the dose chosen as the MTD are (0%, 45.93%) (no DLTs out of six patients), (0.42%, 64.12%) (one DLT out of six patients) and (4.33%, 77.72%) (two DLTs out of six patients). There is a 29% chance that dose level 3 is recommended as the MTD, and a 26% chance that patients in the trial will receive this dose level. If we assume that a 33% DLT probability is to be targeted, then the closest dose is dose level 3, yet there is a 38% chance that dose levels 4 or 5 will be recommended as the MTD. Although the toxicity constraints are more relaxed in this scenario than the previous two, there is approximately a 1% chance that no MTD will be recommended. For this design, the tipping point is 0.448; here the modal MTD is dose level 3, but there may exist other dose-toxicity scenarios where the modal MTD is actually above the tipping point for this design.

### Comparison to pmtd program

The pmtd program, published by Lin and Shih [6], is another available tool that computes operating characteristics for *A* + *B* designs. On a practical basis, the *AplusB* application is far more useful than the pmtd program, since the *AplusB* application does not require software to be downloaded and it is not necessary for the user to have any special programming experience. By comparison, the pmtd program requires the user to download either of the statistical programs R or S-Plus, and also to be familiar with how to install packages and run code in each program.

The *AplusB* application also provides a far more comprehensive analysis of *A* + *B* trial designs than the pmtd program. The output of the pmtd program does not include plots of operating characteristics, and only provides a mean number for some operating characteristics, such as sample size and DLT rate. The *AplusB* application illustrates both the full distribution of the trial sample size and the DLT rate, so clinicians can obtain a thorough understanding of how large their trial is likely to be and what proportion of patients can be expected to experience DLT, even in unlikely scenarios. The *AplusB* application also provides confidence intervals for the probability of DLT at doses that are chosen as the MTD and the tipping point of a design; both of these are related to the trial design rather than a specific dose-toxicity scenario, and so can inform clinicians if using an *A* + *B* design is really the best course of action for their phase I trial. Furthermore, the results provided by *AplusB* can be quickly downloaded as a pre-formatted PDF report so results can easily be presented to other investigators.

With regards to the calculations made by *AplusB* and pmtd, the main difference lies in how the percentage of patients receiving each dose level are calculated. For the pmtd program, the percentage is computed by dividing the average number of patients at a dose by the average number of patients in a trial. The *AplusB* application, however, computes the percentage of patients receiving a particular dose using the probability that each possible trial could occur, therefore giving an exact answer, rather than an approximation. For all four examples, we compared experimentation percentages from *AplusB*, which uses the probability of each trial pathway occurring in its calculations, to those from the pmtd program [6] (Table 1). We found that pmtd overestimates experimentation percentages at higher dose levels by up to 2.1% and underestimates experimentation at lower dose levels by up to 2.4%; this is because pmtd incorrectly gives more weighting to trial pathways that escalate all the way to the maximum planned dose.

**Table 1:** Comparison of experimentation percentages for *AplusB* and pmtd for Examples I, II, III and IV.

Scenario	Method	Dose Level					
		1	2	3	4	5	6
I	*AplusB*	29.8	30.3	29.9	10.0	-	-
	pmtd	27.6	29.4	31.0	12.0	-	-
II	*AplusB*	24.3	24.9	24.5	19.2	6.7	0.3
	pmtd	22.1	23.6	24.6	21.0	8.3	0.4
III	*AplusB*	29.8	32.0	22.9	9.8	4.0	1.4
	pmtd	25.1	30.0	24.3	12.5	5.9	2.3
IV	*AplusB*	20.4	26.7	25.8	18.3	8.8	-
	pmtd	18.0	25.1	26.2	20.4	10.3	-

### Computation times

We calculated mean computation times for generating all trial pathways for *A* + *B* designs with *A* = *B* ∈ {1, …, 6}, with between two and 10 dose levels using *AplusB*; we ran 100 independent runs of each trial design using an AMD Opteron^™^ Processor 6174 2.2GHz running Linux Ubuntu 12.04.5 LTS. For a 6 + 6 trial with {*C*, *D*, *E*} = {1, 1, 1} and 10 dose levels, the mean computation time was 11.352 seconds (standard deviation 2.934 seconds). In general, computation time increases when *C*, *D* and *E* are not all equal and when de-escalation is permitted (see S1, S2, S3 and S4 Tables).

## Discussion

One of the main barriers to increasing the use of new and potentially superior dose-escalation designs in phase I cancer trials has been a lack of user-friendly software [19, 20]. Whilst this is often mentioned in reference to software for new designs that employ statistical models, it is also important that user-friendly software is available for clinicians and investigators to better understand methods that are firmly established in practice. Several papers have shown that because *A* + *B* designs do not target an explicit probability of DLT for the MTD to have, the true probability of DLT at a dose chosen to be the MTD is likely to vary greatly [9, 10]. Furthermore, since the MTD is often chosen based solely on the few patients that have received a particular dose, the uncertainty around the estimated probability of DLT at the MTD will be very large [21]; Chiuzan *et al*. [22] showed that by analysing *A* + *B* designs under a classical statistical framework, there is often too little evidence to select the correct dose as the MTD based on the numbers of patients treated per dose. Many comparative simulation studies have concluded that other rule-based designs and also model-based designs are more likely to recommend correct doses as the MTD than the 3 + 3 design, as well as dose more trial patients at or around the MTD [15, 23–29]. It is important that these aspects of a dose-escalation design are familiar to the investigators, as some designs may be inherently more likely to recommend ineffective doses for phase II testing, and thus increase the chance that a truly superior cancer treatment is shelved due to lack of benefit.

For *A* + *B* designs, particularly the 3 + 3 design, several computer programs have been developed over the last 20 years to summarise how patients are likely to be treated and which doses are likely to be recommended for further testing. However, all of these programs require specific knowledge of advanced statistical software. Also, these programs either provide a very limited set of operating characteristics for a design, or have been lost since publication.

We have created the *AplusB* application using the R package Shiny [14] to provide an up-to-date tool for summarising *A* + *B* dose-escalation designs for phase I cancer trials. The *AplusB* application has a graphical user interface and does not rely on the user having knowledge of specialist statistical software. Comprehensive operating characteristics are provided, both in tabular and graphical form, and can easily be downloaded as a pre-formatted report.

We have released this application on the internet for free (available at http://www.mrc-bsu.cam.ac.uk/software), so that it is easily accessible and more likely to be useful to clinical researchers working in drug development. In addition to this, the code for the AplusB application may also be downloaded from GitHub (https://github.com/graham-wheeler/AplusB) and run remotely on the user’s machine without an internet connection (the R program is required and easy-to-follow setup instructions are provided).

We have demonstrated and discussed how the *AplusB* application is superior to it’s next-best competitor, the S-Plus program pmtd [6], with respect to accessibility, ease of use, accuracy of computation and comprehensiveness of operating characteristics produced. We envisage that this tool will be useful to investigators and clinicians working in phase I cancer trials, both for understanding the implications of using *A* + *B* designs and for comparing the operating characteristics of *A* + *B* designs to other dose-escalation designs, and welcome feedback and comments on its performance.

## Supporting Information

S1 Text Operating characteristics and formulae. A list of operating characteristics provided by the *AplusB* app and their formulae.
(PDF)

S2 Text Relationship between tipping point and MTD selection. Two theorems with proofs on the relationship between the tipping point and the probability of selecting a dose as the MTD.
(PDF)

S1 Table Mean computation times in seconds for *A* + *B* designs. Assumed *A* = *B*, {*C*, *D*, *E*} = {1, 1, 1} and de-escalation is not permitted. *n* = 100.
(PDF)

S2 Table Standard deviation of computation times in seconds for *A* + *B* designs. Assumed *A* = *B*, {*C*, *D*, *E*} = {1, 1, 1} and de-escalation is not permitted. *n* = 100.
(PDF)

S3 Table Mean computation times in seconds for *A* + *B* designs. Assumed *A* = *B*, {*C*, *D*, *E*} = {1, 1, 2} and de-escalation is not permitted. *n* = 100.
(PDF)

S4 Table Standard deviation of computation times in seconds for *A* + *B* designs. Assumed *A* = *B*, {*C*, *D*, *E*} = {1, 1, 2} and de-escalation is not permitted. *n* = 100.
(PDF)
