# Supplementary material for: AplusB: A Web Application for Investigating A + B Designs for Phase I Cancer Clinical Trials
Source: PLoS One. 2016 Jul 12;11(7):e0159026. doi: 10.1371/journal.pone.0159026 (PMC4942070; doi:10.1371/journal.pone.0159026)
Supplement: S1 Text — A list of operating characteristics provided by the AplusB app and their formulae. (PDF) [file pone.0159026.s001.pdf]

S1 Formulae for operating characteristics

S1.1 Trial pathway

A *trial pathway* is defined as the sequence of dose levels each cohort receives, along with the number of patients and number of DLTs per cohort, and the probability of each trial pathway occurring. For any given design, with true DLT probabilities specified, the probability of a particular trial pathway occurring can be expressed as a product of binomial probabilities. For cohort $k \in \{1, ..., K\}$, where K is the maximum number of cohorts one may enrol for a particular $A + B$ design, let p_k be the probability of DLT for the dose level given to cohort k, n_k be the number of patients in cohort k and x_k be the number of DLTs observed in cohort k. Then the probability of trial pathway $\kappa = \{(p_1, n_1, x_1), \dots, (p_K, n_K, x_K)\}$ occurring is

$$\mathbb{P}(\kappa) = \prod_{k=1}^{K} \binom{n_k}{x_k} p_k^{x_k} (1 - p_k)^{n_k - x_k}. \quad (1)$$

Note that if for $1 < k \leq K$ we have $(n_k, x_k) = (0, 0)$, then $\binom{n_k}{x_k} p_k^{x_k} (1 - p_k)^{n_k - x_k} = 1$.

S1.2 Sample size distribution

The expected sample size of a trial using an $A + B$ design, denoted S, is

$$S = \sum_{i=1}^{P} n_i \mathbb{P}(\kappa_i), \quad (2)$$

where P is the total number of possible trial pathways, n_i is the number of people experimented on in trial $i = \{1, \dots, P\}$ and $\mathbb{P}(\kappa_i)$ is the probability of trial pathway κ_i occurring. For a trial of J dose levels using an $A + B$ design, the minimum possible sample size is A and the maximum sample size is $J(A + B)$.

S1.3 MTD recommendation probabilities

The probability that dose level d_j ($j = \{1, \dots, J\}$) is chosen as the MTD is

$$\mathbb{P}(\text{MTD at } d_j) = \sum_{i=1}^{P} \mathbb{1}_{[\text{MTD of } \kappa_i = d_j]} \mathbb{P}(\kappa_i), \quad (3)$$

where the indicator variable $\mathbb{1}_{[\text{MTD of } \kappa_i = d_j]}$ equals 1 if the MTD is d_j for trial pathway κ_i and 0 otherwise.

S1.4 Experimentation probabilities

The probability of a patient being given dose d_j, denoted $\mathbb{P}(\text{Patient given } d_j)$, is

$$\mathbb{P}(\text{Patient given } d_j) = \sum_{i=1}^{P} \mathbb{P}(\text{Patient given } d_j \,|\, \kappa_i)\mathbb{P}(\kappa_i). \tag{4}$$

The results from the *AplusB* application are different to those obtained from the `pmtd` program of Lin and Shih [1]. This is because for the `pmtd` program,

$$\mathbb{P}(\text{Patient given } d_j) \approx \frac{\mathbb{E}(X_j)}{\sum_{j=1}^{J} \mathbb{E}(X_j)} = \frac{\sum_{l=1}^{J} \mathbb{E}(X_j \,|\, \text{MTD } = d_l)\mathbb{P}(\text{MTD } = d_l)}{\sum_{j=1}^{J} \sum_{l=1}^{J} \mathbb{E}(X_j \,|\, \text{MTD } = d_l)\mathbb{P}(\text{MTD } = d_l)}, \tag{5}$$

where X_j is the number of patients receiving dose d_j. This is an approximation that assumes each trial pathway is equally likely to occur. Let n_i be the sample size of trial pathway i and n_{ij} be the number of patients in trial pathway i that receive dose level d_j, so $\sum_{j=1}^{J} n_{ij} = n_i$. We see equation 4 differs from equation 5, since

$$\frac{\mathbb{E}(X_j)}{\sum_{j=1}^{J} \mathbb{E}(X_j)} = \frac{\sum_{i=1}^{P} n_{ij}\mathbb{P}(\kappa_i)}{\sum_{i=1}^{P} n_i\mathbb{P}(\kappa_i)} \neq \frac{\sum_{i=1}^{P} \frac{n_{ij}}{n_i}\mathbb{P}(\kappa_i)}{\sum_{i=1}^{P} \frac{n_i}{n_i}\mathbb{P}(\kappa_i)} = \sum_{i=1}^{P} \mathbb{P}\left(\text{Patient given } d_j \,|\, \kappa_i\right)\mathbb{P}(\kappa_i). \tag{6}$$

S1.5 Expected Toxicity Level (ETL) and Expected Overall Toxicity Rate (EOTR)

The ETL, the expected probability of DLT at the MTD, is

$$\begin{aligned} ETL &= \mathbb{P}(\text{DLT at MTD} \,|\, d_1 \leq \text{MTD } \leq d_J) \\ &= \sum_{j=1}^{J} \mathbb{P}(\text{DLT at MTD} \,|\, \text{MTD } = d_j)\mathbb{P}(\text{MTD } = d_j \,|\, d_1 \leq \text{MTD } \leq d_J) \\ &= \frac{\sum_{j=1}^{J} \mathbb{P}(\text{DLT at } d_j)\mathbb{P}(\text{MTD } = d_j)}{\sum_{l=1}^{J} \mathbb{P}(\text{MTD } = d_l)}. \end{aligned} \tag{7}$$

The inclusion of the possibility that the MTD is equal to the maximum planned dose d_J was excluded by Lin and Shih [1] and Chen *et al.* [2]. They state that if dose-escalation is still indicated at the largest dose under investigation, then the MTD is not determined [1]. This is misleading, since d_J can be recommended as

De-escalation permitted	Data at MTD	Conditions
Yes	δ_1 DLTs out of $A + B$ patients	$0 \leq \delta_1 \leq E$
No	δ_2 DLTs out of A patients	$0 \leq \delta_2 < C$
	δ_3 DLTs out of $A + B$ patients	$C \leq \delta_3 \leq E$

Table S1.1: Possible outcomes observed at the selected MTD from an $A+B$ design, with and without de-escalation.

the MTD in practice; therefore, the formula above is used in *AplusB*. The Expected Overall Toxicity Rate (EOTR), is defined to be the expected number of DLTs divided by the expected number of patients, i.e.

$$EOTR = \frac{\sum_{j=1}^{J} \mathbb{P}(\text{DLT at } d_j)\mathbb{E}(X_j)}{\sum_{j=1}^{J} \mathbb{E}(X_j)}. \tag{8}$$

S1.6 Data at trial end and confidence intervals

At the end of a trial that follows an $A + B$ design, we may calculate $100(1 - \alpha)\%$ confidence intervals for the estimate of the probability of DLT at the identified MTD. The data at the MTD determined at the end of the trial will be dependent on whether dose-escalation is permitted or not (Table S1.1). Clopper-Pearson confidence intervals [3] and Wilson score confidence intervals [4] are provided; exact confidence intervals are conservative intervals derived directly from the Binomial distribution, whereas Wilson score intervals provide better coverage and may be more suitable for constructing intervals based on small samples [5–7].

S1.7 Tipping Point

The *tipping point* is the true DLT probability a dose must have at which the chance of escalating to the next dose level is equal to the chance de-escalating or stopping the trial. For a general $A + B$ design, the probability of escalating from d_j to d_{j+1} is

$$
\begin{aligned}
\mathbb{P}(\text{Escalate from } d_j \text{ to } d_{j+1}) &= \mathbb{P}(Y_j^A < C) + \sum_{v=C}^{D} \mathbb{P}(Y_j^A = v)\mathbb{P}(Y_j^B \leq E - v) \\
&= \sum_{0 \leq u < C} \binom{A}{u} p_j^u (1 - p_j)^{A-u} + \sum_{v=C}^{D} \binom{A}{v} p_j^v (1 - p_j)^{A-v} \sum_{0 \leq w \leq E-v} \binom{B}{w} p_j^w (1 - p_j)^{B-w}
\end{aligned}
\tag{9}
$$

where Y_j^A and Y_j^B are the number of DLTs observed after dosing A and B patients at dose d_j respectively. To find the tipping point, we set $\mathbb{P}(\text{Escalate from } d_j \text{ to } d_{j+1}) = 0.50$ and solve for p_j using numerical methods; the real solution to this equation that lies between 0 and 1 is the tipping point.

References

[1] Lin Y, Shih WJ. Statistical properties of the traditional algorithm-based designs for phase I cancer clinical trials. Biostatistics. 2001;2(2):203–215.

[2] Chen Z, Krailo MD, Sun J, Azen SP. Range and trend of expected toxicity level (ETL) in standard A + B designs: a report from the Children's Oncology Group. Contemporary Clinical Trials. 2009;30(2):123–128.

[3] Clopper CJ, Pearson ES. The use of confidence or fiducial limits illustrated in the case of the binomial. Biometrika. 1934;26(4):404–413.

[4] Wilson EB. Probable inference, the law of succession, and statistical inference. Journal of the American Statistical Association. 1927;22:209–212.

[5] Agresti A, Coull BA. Approximate is better than "exact" for interval estimation of Binomial proportions. The American Statistician. 1998;52(2):119–126.

[6] Agresti A, Caffo B. Simple and effective confidence intervals for proportions and differences of proportions result from adding two successes and two failures. The American Statistician. 2000;54(4):280–288.

[7] Brown LD, Cai TT, DasGupta A. Interval Estimation for a Binomial Proportion. Statistical Science. 2001;16(2):101–133.
