# Supplementary material for: AplusB: A Web Application for Investigating A + B Designs for Phase I Cancer Clinical Trials
Source: PLoS One. 2016 Jul 12;11(7):e0159026. doi: 10.1371/journal.pone.0159026 (PMC4942070; doi:10.1371/journal.pone.0159026)
Supplement: S2 Text — Two theorems with proofs on the relationship between the tipping point and the probability of selecting a dose as the MTD. (PDF) [file pone.0159026.s002.pdf]

S2 Relationship between tipping point and MTD selection

Here we present two theorems that relate the tipping point to how likely a dose is to be chosen as an MTD under particular design.

Theorem 1. *For any $A + B$ design on $J \geq 2$ doses, let $\hat{p}$ be its tipping point. The probability of choosing a dose with true DLT probability equal to or above $\hat{p}$ as the MTD is at most* 50%.

Proof. Let d_j be the first dose level with true DLT probability equal to or higher than $\hat{p}$. By definition of the tipping point, the chance that we de-escalate to dose d_{j-1} or terminate the trial at d_j is at least 50%, and the chance we escalate to d_{j+1} is at most 50%. If we terminate the trial at d_j, then we declare d_{j-1} as the MTD and if we de-escalate to d_{j-1}, we will never declare a dose level higher than d_{j-1} as the MTD; therefore, the chance of choosing any dose level with true DLT probability equal to or above $\hat{p}$ as the MTD is at most 50%, and so we are done. □

Theorem 2. *For an $A + B$ design without dose de-escalation on $J \geq 2$ doses, the dose most likely to be chosen as the MTD has a true DLT probability of at most $\hat{p}$.*

Proof. Let p_j denote the true DLT probability at dose d_j. For the case where all doses d_j ($j = \{1, \ldots, J\}$) have true DLT probability below $\hat{p}$, one of these doses has to be the most common and so we are done. Let us assume at least one d_j has a true DLT probability above $\hat{p}$, i.e. $p_{j-1} \leq \hat{p} < p_j$ for $j \geq 1$ ($j = 1$ implies all dose levels in the trial have true DLT probabilities above $\hat{p}$, with p_0 being the probability of no dose (d_0) being selected as the MTD due to severe toxicity). Then the probability of declaring d_{j-1} or some lower dose as the MTD is greater than 0.50 (by Theorem 1). If $j = 1$, then we are done, since the trial would terminate and the most likely MTD choice would be d_0. For $j > 1$, we have at least a 50% chance of stopping the trial at d_j (since $\hat{p} < p_j$), which implies that we are more likely to choose d_{j-1} as the MTD than d_j, and so we are done. □
