# Supplementary material for: AplusB: A Web Application for Investigating A + B Designs for Phase I Cancer Clinical Trials
Source: PLoS One. 2016 Jul 12;11(7):e0159026. doi: 10.1371/journal.pone.0159026 (PMC4942070; doi:10.1371/journal.pone.0159026)
Supplement: S1 Table — Assumed A = B, {C, D, E} = {1, 1, 1} and de-escalation is not permitted. n = 100. (PDF) [file pone.0159026.s003.pdf]

$A = B$	Number of dose levels								
	2	3	4	5	6	7	8	9	10
1	0.139	0.269	0.525	0.992	1.459	2.212	3.160	4.553	6.324
2	0.139	0.284	0.565	0.969	1.518	2.311	3.375	4.912	7.264
3	0.117	0.294	0.559	0.964	1.544	2.322	3.554	5.414	8.719
4	0.126	0.290	0.568	0.980	1.554	2.428	3.716	5.903	9.930
5	0.121	0.291	0.567	0.987	1.605	2.514	3.931	6.421	10.636
6	0.124	0.301	0.588	0.993	1.623	2.559	4.124	6.890	11.352

Table S1: Mean computation times in seconds for $A + B$ designs. Assumed $A = B$, $\{C, D, E\} = \{1, 1, 1\}$ and de-escalation is not permitted. $n = 100$.
