# Supplementary material for: AplusB: A Web Application for Investigating A + B Designs for Phase I Cancer Clinical Trials
Source: PLoS One. 2016 Jul 12;11(7):e0159026. doi: 10.1371/journal.pone.0159026 (PMC4942070; doi:10.1371/journal.pone.0159026)
Supplement: S2 Table — Assumed A = B, {C, D, E} = {1, 1, 1} and de-escalation is not permitted. n = 100. (PDF) [file pone.0159026.s004.pdf]

$A = B$	Number of dose levels								
	2	3	4	5	6	7	8	9	10
1	0.054	0.044	0.081	0.156	0.243	0.340	0.452	0.836	1.564
2	0.054	0.046	0.087	0.139	0.255	0.344	0.512	0.981	1.861
3	0.021	0.057	0.086	0.142	0.251	0.351	0.587	1.268	2.286
4	0.035	0.055	0.088	0.160	0.255	0.388	0.647	1.483	2.509
5	0.023	0.049	0.085	0.167	0.263	0.402	0.720	1.684	2.735
6	0.025	0.045	0.100	0.153	0.255	0.406	0.815	1.864	2.934

Table S2: Standard deviation of computation times in seconds for $A + B$ designs. Assumed $A = B$, $\{C, D, E\} = \{1, 1, 1\}$ and de-escalation is not permitted. $n = 100$.
