# Supplementary material for: AplusB: A Web Application for Investigating A + B Designs for Phase I Cancer Clinical Trials
Source: PLoS One. 2016 Jul 12;11(7):e0159026. doi: 10.1371/journal.pone.0159026 (PMC4942070; doi:10.1371/journal.pone.0159026)
Supplement: S3 Table — Assumed A = B, {C, D, E} = {1, 1, 2} and de-escalation is not permitted. n = 100. (PDF) [file pone.0159026.s005.pdf]

$A = B$	Number of dose levels								
	2	3	4	5	6	7	8	9	10
1	0.371	0.866	1.227	1.248	1.971	2.826	5.630	13.546	33.324
2	0.331	0.931	1.617	1.935	3.141	4.721	11.936	28.015	80.167
3	0.263	0.776	1.301	1.878	2.927	5.505	15.280	37.024	115.234
4	0.256	0.731	1.143	1.922	2.981	6.715	17.260	43.238	149.624
5	0.365	0.788	1.040	1.942	3.238	8.342	19.908	50.919	180.067
6	0.469	0.871	1.046	2.014	3.368	8.761	22.127	58.944	214.449

Table S3: Mean computation times in seconds for $A + B$ designs. Assumed $A = B$, $\{C, D, E\} = \{1, 1, 2\}$ and de-escalation is not permitted. $n = 100$.
