# Supplementary material for: AplusB: A Web Application for Investigating A + B Designs for Phase I Cancer Clinical Trials
Source: PLoS One. 2016 Jul 12;11(7):e0159026. doi: 10.1371/journal.pone.0159026 (PMC4942070; doi:10.1371/journal.pone.0159026)
Supplement: S4 Table — Assumed A = B, {C, D, E} = {1, 1, 2} and de-escalation is not permitted. n = 100. (PDF) [file pone.0159026.s006.pdf]

$A = B$	Number of dose levels								
	2	3	4	5	6	7	8	9	10
1	0.992	1.555	2.529	1.250	2.006	2.351	4.196	12.662	16.828
2	0.728	1.652	3.181	2.637	3.175	3.748	13.136	21.569	58.720
3	0.357	1.521	2.291	1.927	2.606	4.114	16.283	28.110	85.627
4	0.326	1.283	1.226	2.102	3.230	4.440	16.302	24.079	117.398
5	0.817	1.484	0.915	2.100	3.746	8.969	18.729	30.526	130.481
6	1.008	1.568	0.913	2.050	2.589	6.771	18.137	28.143	141.181

Table S4: Standard deviation of computation times in seconds for $A + B$ designs. Assumed $A = B$, $\{C, D, E\} = \{1, 1, 2\}$ and de-escalation is not permitted. $n = 100$.
